# *Neural EGFL like 1* as a novel gene for Trabecular Bone Score in older adults: The Bushehr Elderly Health (BEH) program

**DOI:** 10.1371/journal.pone.0309401

**Published:** 2024-09-10

**Authors:** Mohammad Bidkhori, Mahdi Akbarzadeh, Noushin Fahimfar, Mina Jahangiri, Sahar Seddiq, Bagher Larijani, Iraj Nabipour, Mahsa Mohammad Amoli, Nekoo Panahi, Abbas Dehghan, Kourosh Holakouie-Naieni, Afshin Ostovar

**Affiliations:** 1 Department of Epidemiology and Biostatistics, School of Public Health, Tehran University of Medical Sciences, Tehran, Iran; 2 Osteoporosis Research Center, Endocrinology and Metabolism Clinical Sciences Institute, Tehran University of Medical Sciences, Tehran, Iran; 3 Cellular and Molecular Endocrine Research Center, Research Institute for Endocrine Sciences, Shahid Beheshti University of Medical Sciences, Tehran, Iran; 4 Department of Biostatistics, Faculty of Medical Sciences, Tarbiat Modares University, Tehran, Iran; 5 Endocrinology and Metabolism Research Center, Endocrinology and Metabolism Clinical Sciences Institute, Tehran University of Medical Sciences, Tehran, Iran; 6 The Persian Gulf Marine Biotechnology Research Center, The Persian Gulf Biomedical Sciences Research Institute, Bushehr University of Medical Sciences, Bushehr, Iran; 7 Metabolic Disorders Research Center, Endocrinology and Metabolism Molecular -Cellular Sciences Institute, Tehran University of Medical Sciences, Tehran, Iran; 8 Department of Biostatistics and Epidemiology, MRC-PHE Centre for Environment and Health, School of Public Health, Imperial College London, London, United Kingdom; Nanjing Medical University, CHINA

## Abstract

*Neural EGFL like 1 (NELL-1*), is a secreted glycoprotein and stimulates osteogenic cell differentiation and bone mineralization. This study aimed to explore the relationship between *NELL-1* and Trabecular Bone Score (TBS) as a novel tool for the evaluation of osteoporosis in an elderly population-based cohort study in Iran. A single-locus analysis was performed on TBS using data from 2,071 participants in the Bushehr Elderly Health (BEH) Program. The study investigated 376 independent single nucleotide polymorphisms (SNPs) within the *NELL-1* on chromosome 11p15.1. The association between SNPs and the mean TBS L1 to L4 was analyzed through an additive model. Significant variants in the additive model (P_FDR_<0.05) were further examined within dominant, recessive, over-dominant, and co-dominant models. Multiple linear regression was employed to assess the relationship between the genetic risk score (GRS) derived from significant SNPs and TBS. Three SNPs within the *NELL-1* showed a statistically significant association with TBS after adjusting for age and sex. The associations for rs1901945 (β = 0.013, P_FDR_ = 0.0007), rs1584851 (β = -0.011, P_FDR_ = 0.0003), and rs58028601 (β = 0.011, P_FDR_ = 0.0003) were significant in the additive model. Additionally, significant results were observed for rs1901945 and rs58028601 in the dominant model (P<0.05). The GRS showed a statistically significant relationship with TBS, considering adjustments for age, sex, Body Mass Index, type 2 diabetes, and smoking (β = 0.077, P = 1.7×10^−5^). This study highlights the association of *NELL-1* with TBS, underscoring its potential as a candidate for further research and personalized medicine concerning the impact of this gene on bone quality.

## Introduction

Osteoporosis is a systemic skeletal disease defined as a bone mineral density (BMD) 2.5 Standard deviation or more below that of young, healthy women in lumbar spine, total hip, or femoral neck assessed by DXA [1, 2]. The global burden of osteoporosis is increasing; its Worldwide prevalence in women and men is estimated at 23.1% and 11.7%, respectively [3, 4]. The age-standardized prevalence of osteoporosis among Iranian men and women aged 60 years or older, was 24.6% and 62.7%, respectively [5]. Additionally, the estimated annual cumulative incidence of hip fractures within this population is reported at 138 per 100,000 in men and 157 per 100,000 in women [6]. The economic ramifications of osteoporosis are substantial, with the economic burden in Iran reaching an estimated US$ 393.24 million in 2020 [7].

A significant number of osteoporotic fractures occur in individuals with normal BMD scores, indicating that factors beyond bone density, such as bone microarchitecture, play a significant role in fracture risks [8–11]. The Trabecular Bone Score (TBS) is a novel and complementary index that evaluates bone microarchitecture based on pixel variation in gray levels in the lumbar spine dual-energy X-ray absorptiometry (DXA) image [12–18]. TBS and BMD are highly correlated and share similar heritability with genetic factors accounting for around 45% of the variance in TBS. This indicates that genetics play an important role in determining both bone mineral density and microarchitecture [19]. A low TBS is related to an increased fracture risk, independent of BMD, and combining these two parameters improves the fracture risk prediction process [14–17].

The development of osteoporosis results from the interaction between environmental, metabolic and genetic factors, each exerting modest effects on bone metabolism and fracture susceptibility [20–24]. Although osteoporosis is in the early stages of personalized medicine implementation, polygenic risk scores based on multiple genetic variants have allowed for the assessment of an individual’s genetic susceptibility to the condition [25, 26].

*Neural EGFL like 1 (NELL-1)* is a protein-coding gene that encodes a cytoplasmic protein and is involved in regulating cell growth and differentiation [27]. *NELL-1* promotes osteoblast cell and osteoblastic mineralization and proliferation [28]. Recent studies have revealed the role of *NELL-1* in bone health. Researchers have found, for the first time, that NELL-1 expression is increased in cranial intramembranous bone from patients with craniosynostosis [29]. Several investigations have consistently shown that *NELL-1* plays a crucial role in the osteogenic differentiation of osteoblasts, and it has been found to interact synergistically with other genes implicated in the development of osteoporosis, such as Bone Morphogenetic Protein 2 (BMP2) [30–32]. NELL-1 has been identified as a promising therapeutic target for treating osteoporosis in animal studies. The findings revealed that the groups treated with NELL-1 indicated increased bone formation and enhanced new bone growth compared to the control group [33, 34].

Genetic risk factors can be varied across different human populations [35]. Given the novelty of TBS, no prior studies have investigated genes potentially influencing TBS in the Iranian population. The investigation of genetic loci associated with TBS is crucial for personalized medicine, specifically in identifying high-risk elderly individuals at high risk with low TBS. Our study was the first to demonstrate the genetic risk of *NELL-1* on TBS within the Iranian population.

## Materials and methods

### Participants

The present study was conducted on 2,071 participants of the Bushehr Elderly Health (BEH) study. This population-based prospective cohort study initiated in 2013, comprises a total of 3,000 adults, including 1,000 men (48.2%), all aged 60 years or older, selected through a multistage cluster random sampling method [36]. The main objective of the second measurement of the first phase was to investigate musculoskeletal health and the related risk factors and consequences in 2,772 participants who were under follow-up in 2015 [37].

### Genotyping and quality control

Blood samples obtained from the participants in the BEH study were genotyped using the Infinium Global Screening Array (GSA) from Illumina. A total of 47,109,443 autosomal SNPs were imputed, and the quality of the imputation was assessed by means of MACH R-square. A systematic and comprehensive approach was adopted to implement genomic quality control (QC) [38]. Good quality was defined as a MACH R-square above 0.3 and SNPs with a Hardy–Weinberg equilibrium P<10^−6^, and minor allele frequency ≤0.01 were excluded from the analysis. All SNPs and gene locations were relative to the GRCh37 genome assembly.

### Gene selection

The *NELL-1* is a protein-coding gene located on chromosome 11 (11p15.1), with its genomic region spanning from position 20,691,097 to 21,597,232 (GRCh37) on the chromosome (27). To ensure comprehensive coverage of potential primer regions, we considered a genomic region spanning approximately ±5 kb surrounding the gene. Following quality control procedures, the dataset contained 3,451 SNPs related to the *NELL-1* gene. Out of the extracted SNPs, 376 independent SNPs with a low linkage disequilibrium (r^2^≤0.2) were selected.

### Outcome measures

The Trabecular Bone Score (TBS) of each lumbar spine was assessed using TBS iNsight® software version 2.2, installed on a dual X-ray absorptiometry (DXA) machine (Discovery WI, Hologic Inc., USA). The mean L1 to L4 lumbar spine TBS was considered as the outcome of this study. Higher TBS values indicate a more robust trabecular structure, whereas lower TBS values are associated with an increased risk of osteoporosis and osteoporotic fractures.

### Statistical analysis

A generalized linear model (GLM) was applied to examine various genetic models, including additive, dominant, recessive, over-dominant and co-dominant models. Initially, an analysis was conducted on the 376 *NELL-1* gene variants to explore their relationship with TBS using the additive model, with a significance threshold set at a False Discovery Rate (P_FDR_) <0.05. Subsequently, SNPs identified as significant in the additive model (P_FDR_ <0.05) were further evaluated across dominant, recessive, over-dominant, and co-dominant models. Different genetic models for a SNP were defined as demonstrated in the following example. For rs1901945 with alleles C/G, where C is the minor allele, the coding labels for the additive model were defined based on the minor allele count as GG = 0, CG = 1, and CC = 2. Additionally, the dominant model (CC + CG versus GG), recessive model (CC versus CG + GG), co-dominant model (CC and CG versus GG), and over-dominant model (CG versus CC + GG) were investigated.

A genetic risk score (GRS) was calculated for each individual through regression coefficients and allele values derived from significant genetic variants. To calculate the GRS for an individual, we assigned values to the alleles based on whether or not it is the effect allele (0, 1, and 2). Then, we multiplied these allele values by the corresponding beta coefficients for each genetic variant. The sum of these products provides the GRS for that individual. The relationship between GRS and TBS was investigated using simple and multiple linear regression analyses, adjusting for age, sex, Body Mass Index (BMI), type 2 diabetes and smoking status. Associations between GRS quartiles and TBS were also investigated by Analysis of Variance (ANOVA).

A volcano plot was utilized to visually represent the results of the additive model for all the 376 investigated SNPs. This plot typically displays the negative logarithm (base 10) of the p-value on the y-axis and regression coefficients on the x-axis for each SNP. Each point on the plot corresponds to a specific SNP. The interpretation involves assessing the position of points relative to the axes. SNPs positioned higher on the y-axis signify a stronger relationship with TBS, while those located towards the edges on the x-axis indicate larger expression differences, suggesting biological relevance.

The statistical significance threshold for the additive model was considered to be P_FDR_<0.05. Additionally, the threshold for the GRS was set at P<0.05.

Quality control processes were performed using PLINK version 2 software, and the relationships of SNPs with TBS in different genetic models were investigated using R version 4.4.0 (SNPassoc package) [39]. In addition, simple and multiple linear regression models, as well as data visualization were conducted using R version 4.4.0.

### Ethics approval and consent to participate

This study was approved by the Research Ethics Committee of the Tehran University of Medical Sciences under code IR.TUMS.SPH.REC.1400.237. Participants were recruited in the BEH Program only after obtaining written informed consent. Data was accessed for research purposes on November 9, 2021. Authors did not have access to information that could identify individual participants during or after data collection.

## Results

The participants’ characteristics are presented in Table 1. Among the participants, 51.7% were women, and the average age was 69.4 years. The mean BMI among older adults was 27.53±4.87, with 31.3% of them having been diagnosed with Type 2 diabetes. The mean Trabecular Bone Score (TBS) for the lumbar spine region (L1-L4) was 1.296 ± 0.105 across both sexes. Specifically, the mean TBS values for men and women were 1.353 ± 0.091 and 1.241 ± 0.087, respectively. Additionally, the mean TBS values for the individual lumbar segments (L1, L2, L3, and L4) were estimated as follows: 1.261 ± 0.132, 1.310 ± 0.122, 1.315 ± 0.116, and 1.299 ± 0.125, respectively.

**Table 1:** Baseline and TBS characteristics of study population.

Variable	Men N = 1000 (48.3%)	Women N = 1071(51.7)	Total N = 2071
Age, Mean (SD*) (yr)	69.6 (6.4)	69.2 (6.4)	69.4 (6.4)
BMI, Mean (SD) (kg/m2)	26.33 (3.97)	28.65 (5.34)	27.53 (4.87)
Type 2 Diabetes, Frequency (%)			
Yes	274 (27.6)	371 (34.8)	645 (31.3)
No	720 (72.4)	694 (65.2)	1414 (68.7)
Smoking status, Frequency (%)			
Never	417 (42)	500 (46.9)	917 (44.5)
Current smoker	346 (34.8)	374 (35)	720 (34.9)
Former smoker	231 (23.2)	193 (18.1)	424 (20.6)
TBS L1**, Mean (SD)	1.312 (0.120)	1.213 (0.124)	1.261 (0.132)
TBS L2, Mean (SD)	1.364 (0.108)	1.258 (0.111)	1.31 (0.122)
TBS L3, Mean (SD)	1.374 (0.103)	1.258 (0.098)	1.315 (0.116)
TBS L4, Mean (SD)	1.364 (0.102)	1.236 (0.112)	1.299 (0.125)
TBS L1-L4, Mean (SD)	1.353 (0.091)	1.241 (0.087)	1.296 (0.105)

SD: Standard deviation, TBS: Trabecular bone score

**Fig 1:**
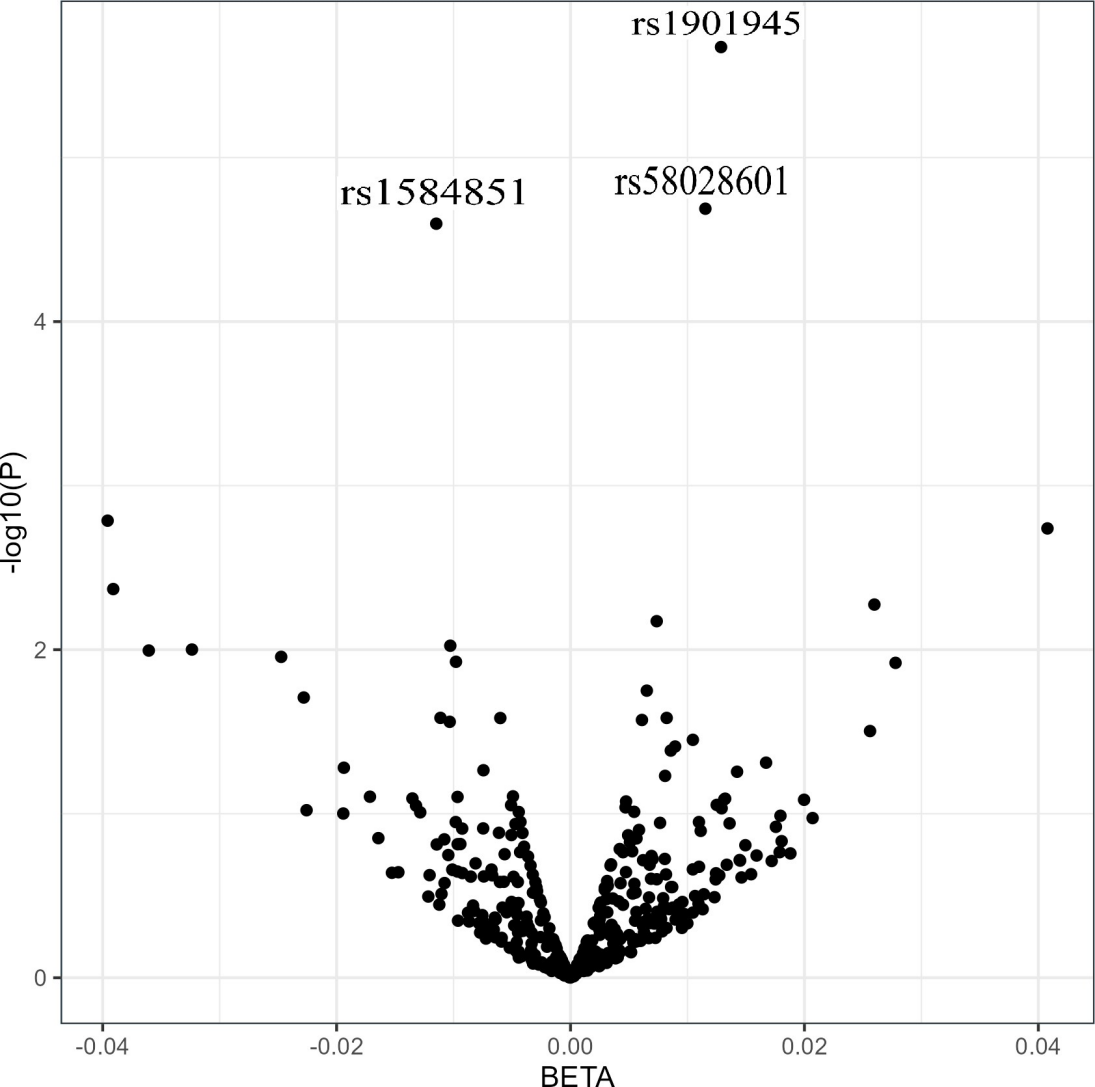
Volcano plot of the relationship between 376 NELL-1 SNPs and trabecular bone score. The x-axis represents the -log10 of the p-value. The y-axis represents the regression coefficients, reflecting the magnitude and direction of the effect size.

Fig 1 displays a volcano plot representing the p-values versus regression coefficients of 376 analyzed SNPs. From the analysis, three SNPs were identified to be significantly associated with TBS adjusted for age and sex in the additive model. The specific characteristics of these significant SNPs are presented in Table 2.

**Table 2:** Characteristics of the statistically significant genetic variants of *NELL-1* related to trabecular bone score.

rsID	Position (GRCh37)	Allele1/Allele2	MAF*	Functional consequence
rs1901945	21285923	C/G	0.48	Intron
rs1584851	21301343	T/C	0.49	Intron
rs58028601	21312971	A/G	0.48	Intron

*Minor Allele Frequency

The relationships between the genetic variants rs1901945-C (β = 0.013, P = 2.12×10^−6^, P_FDR_ = 0.0007), rs1584851- T (β = -0.011, P = 2.52×10^−5^, P_FDR_ = 0.0003), and rs58028601-A (β = 0.11, P = 2.04×10^−5^, P_FDR_ = 0.0003), with TBS were found to be statistically significant in the additive model. For rs1901945-C, each copy of the C allele, on average, increased the TBS by 0.013. Further analyses in different genetic models revealed additional insights: in the dominant model, two SNPs, rs1901945-C (β = 0.02, P = 2.73×10^−6^, P_FDR_ = 0.001), and rs58028601-A (β = 0.018, P = 4.47×10^−5^, P_FDR_ = 0.008) showed a statistically significant association with TBS. As a result, for rs1901945-C, compared to the GG genotype, having one or two copies of the C allele increased TBS by 0.02. Under the co-dominant model, rs1901945-C was related to TBS. For this variant, CG (β = 0.017, P = 9×10–5, P_FDR_ = 0.001) and CC (β = 0.025, P = 3.24×10–6, P_FDR_ = 0.0008) genotypes were statistically significant compared to GG. Significant results for rs1584851-T and rs58028601-A were observed only for the homozygous genotypes with two effect alleles. No significant relationships between the variants and TBS were found in the recessive and over-dominant genetic models (P_FDR_ >0.05) (Table 3).

**Table 3:** Association of genetic variants with the Trabecular bone score, adjusted for age and sex.

rsID	Effect allele	Beta*	CI, 95%*	P-value	P_FDR_ ***
**Additive**					
rs1901945(C/G)	C	0.013	(0.008, 0.018)	2.12×10^−6^	**0.0007**
rs1584851(T/C)	T	-0.011	(-0.017, -0.006)	2.52×10^−5^	**0.003**
rs58028601(A/G)	A	0.011	(0.006, 0.017)	2.04×10^−5^	**0.003**
**Dominant**					
rs1901945(C/G)	C	0.02	(0.01, 0.3)	2.73e×10^−6^	**0.001**
rs1584851(T/C)	T	-0.016	(-0.024, -0.007)	0.0004	0.055
rs58028601(A/G)	A	0.018	(0.009, 0.026)	4.47×10^−5^	**0.008**
**Recessive**					
rs1901945(C/G)	C	0.013	(0.005, 0.023)	0.002	0.2
rs1584851(T/C)	T	-0.015	(-0.024, -0.006)	0.0007	0.2
rs58028601(A/G)	A	0.013	(0.004, 0.022)	0.004	0.3
**Over-dominant**					
rs1901945(C/G)	C	0.006	(-0.001, 0.014)	0.1	0.7
rs1584851(T/C)	T	-0.001	(-0.008, 0.007)	0.8	0.99
rs58028601(A/G)	A	0.005	(-0.003, 0.012)	0.2	0.9
**Co-dominant (Reference)**					
rs1901945 (GG)					
CG		0.017	(0.008, 0.026)	9×10^−5^	**0.001**
CC		0.025	(0.014, 0.035)	3.24×10^−6^	**0.0008**
rs1584851 (CC)					
TC		-0.011	(-0.021, -0.002)	0.01	0.8
TT		-0.02	(-0.033, -0.012)	2.50×10^−5^	**0.001**
rs58028601 (GG)					
AG		0.015	(0.006, 0.024)	0.001	0.3
AA		0.022	(0.012, 0.033)	2.7×10^−5^	**0.0002**

*Adjusted for age and sex

**95% Confidence interval

***False discovery rate

The risk score for the significant SNPs was computed based on the number of risk alleles present, taking into account the respective regression coefficients. The risk scores of all three SNPs were associated with TBS (P<0.05). The results of both simple and multiple linear regression analyses can be found in Table 4.

**Table 4:** Relationship between SNP risk scores and the trabecular bone score.

		Simple linear regression			Multiple linear regression*		
	Effect allele	Standardized Coefficient	CI, 95%*	P-value	Beta	CI, 95%	P-value
Risk score for the additive model							
rs1901945 (C/G)	C	0.092	(0.049, 0.135)	**2.9×10** ^**−5**^	0.082	(0.047, 0.117)	**5.0×10** ^**−6**^
rs1584851 (T/C)	T	0.088	(0.045, 0.131)	**6.5×10** ^**−5**^	0.073	(0.038, 0.108)	**5.2×10** ^**−5**^
rs58028601 (A/G)	A	0.084	(0.041, 0.127)	**1.4×10** ^**−4**^	0.071	(0.036, 0.106)	**8.3×10** ^**−5**^
Risk score for the dominant model							
rs1901945 (C/G)	C	0.096	(0.053, 0.139)	**1.2×10** ^**−5**^	0.083	(0.048, 0.118)	**3×10** ^**−6**^
rs1584851 (T/C)	T	0.068	(0.025, 0.111)	**0.001**	0.061	(0.025, 0.096)	**6.1×10** ^**−4**^
rs58028601 (A/G)	A	0.095	(0.052, 0.138)	**1.3×10** ^**−5**^	0.07	(0.035, 0.105)	**9.9×10** ^**−5**^

†Adjusted for age, sex, BMI, type 2 diabetes and smoking status

*95% Confidence interval

**Fig 2:**
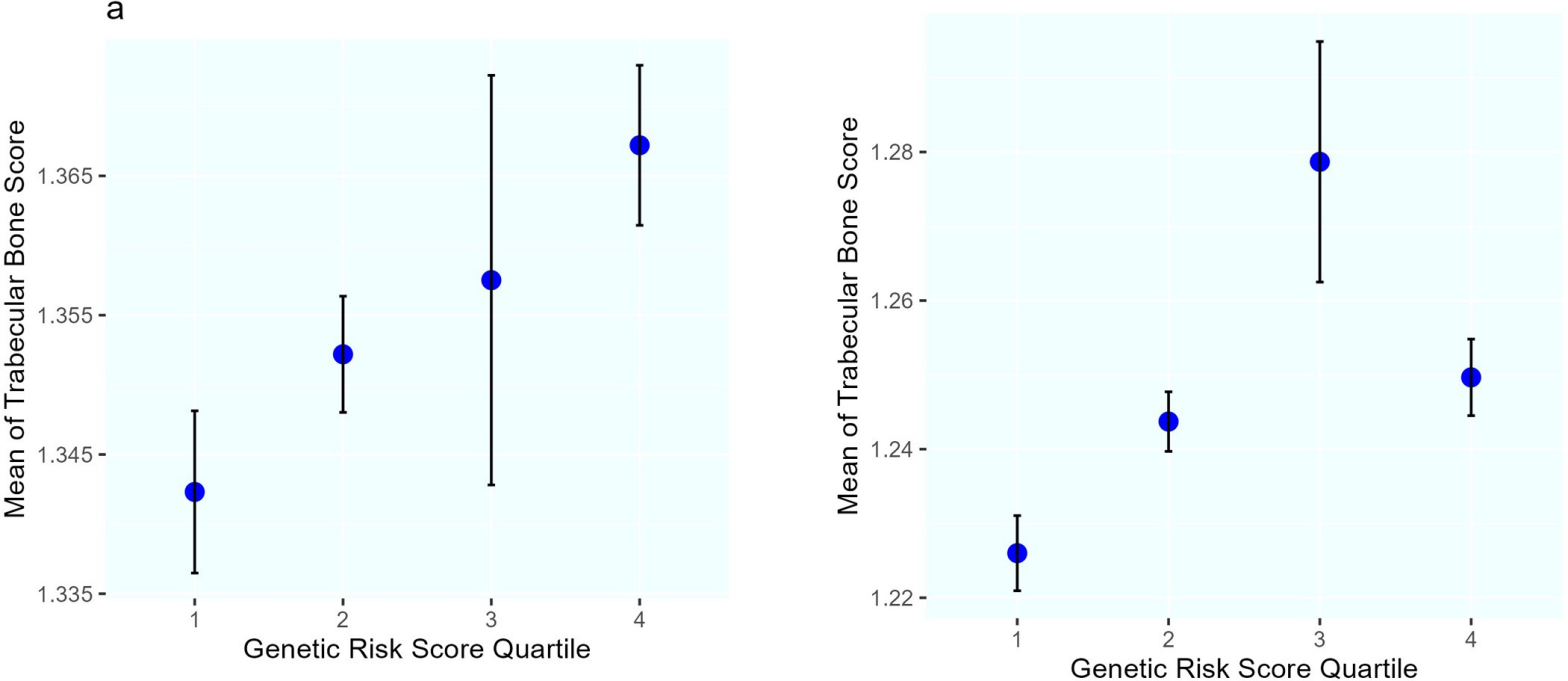
Mean trabecular bone scores (95% confidence interval for the standard error) among quartile categories of the genetic risk score derived from the additive model in a) men and b) women.

In the subsequent analysis, a Genetic Risk Score (GRS) was calculated by summing up the effect of the three SNPs related to TBS. The Correlation between risk scores derived from the additive genetic model (r = 0.09, P = 4×10^−5^) and the dominant genetic model (r = 0.1, P = 4×10^−6^) with TBS was found to be statistically significant. Further exploration involved investigating the associations of GRS quartiles in both additive and dominant models with TBS using ANOVA. The differences between TBS means were statistically significant in four quartile groups of GRS in both men and women (P<0.05). Fig 2 presents the mean TBS in quartiles of GRS for men and women, illustrating an increasing trend across the GRS quartiles. It is noted that the higher mean TBS in the third quartile compared to the fourth in women could be attributed to the smaller sample size, as indicated by the confidence intervals.

Standardized regression coefficients were used to investigate the effect size corresponding to GRS for TBS in older adults. The GRS derived from the additive model showed a statistically significant relationship with TBS even after adjusting for age, sex, BMI, type 2 diabetes, and smoking status. The results indicated that a one standard deviation increase in the GRS corresponds to a 0.086 standard deviation increase in TBS (p = 2×10–6). Similarly, the GRS obtained from the dominant model also demonstrated a significant relationship with TBS (β = 0.086, P = 2×10–6) (Table 5).

**Table 5:** Relationship between genetic risk score (weighted sum scores of risk alleles) and TBS.

	Additive genetic model				Dominant genetic model			
Genetic risk score	Standardized Coefficient	CI, 95%*	P-value	Adjusted R-Square	Standardized Coefficient	CI, 95%	P-value	R-Square
Model 1	0.09	(0.047, 0.133)	**4×10** ^**−5**^	0.008	0.1	(0.062, 0.148)	**2×10** ^**−6**^	0.01
Model 2	0.083	(0.047, 0.119)	**5×10** ^**−6**^	0.31	0.091	(0.055, 0.126)	**7.02×10** ^**−7**^	0.31
Model 3	0.077	(0.042, 0.112)	**1.7×10** ^**−5**^	0.34	0.086	(0.051, 0.126)	**2×10** ^**−6**^	0.34
Model 4	-	-	-	0.30	-	-	-	0.30
Model 5	-	-	-	0.33	-	-	-	0.33

Model 1: Crude, Model 2: Age, sex, and **GRS**, Model 3: Age, sex, BMI, type 2 diabetes, smoking status, and **GRS**, Model 4: Age, sex, Model 5: Age, sex, BMI, type 2 diabetes, and smoking status

*95% Confidence interval

Furthermore, adjusted R-squared (R^2^) values were calculated for various regression models to evaluate the proportion of the variance in TBS explained by the independent variables. The R^2^ values for the five regression models, including: 1) the crude model, 2) the model with Age, sex, and GRS, 3) Age, sex, BMI, type 2 diabetes, smoking status, and GRS, Model, 4) Age, sex, 5) Age, sex, BMI, type 2 diabetes, and smoking status, were determined to be 0.008, 0.31, 0.34, 0.3, and 0.339, respectively. It was observed that the models incorporating GRS showed a slight increase in R2 values compared to the models without GRS (Model 2 vs Model 4 and Model 3 vs Model 5).

## Discussion

The Current study has identified a relationship between TBS and three specific SNPs located in the *NELL-1* gene (rs1901945, rs1584851, and rs58028601) under the additive, dominant, and co-dominant genetic models.

*NELL-1* was found to be associated with TBS in our study. Only a limited number of studies have been conducted to identify genes that influence TBS. Among the genes that have been found to be related to TBS are *GREM2* and *CYP24A1* [40, 41], while a genome-wide association study identified two loci near *IRX3* and *MAP2K5* that may also have a role in TBS [42]. Given that TBS is a relatively recent tool, the absence of prior reports of *NELL-1* as a gene associated with TBS is not unexpected. Nonetheless, numerous studies in recent decades have demonstrated the functional impact of *NELL-1* on bone formation.

Previous studies have identified the functional impact of the *NELL-1* gene on bone health. Ting et al. in 1999, showed that *NELL-1* is expressed and up-regulated in cranial intramembranous bone in craniosynostosis patients [29]. A study conducted in 2006 revealed that decreased expression of *NELL-1* affected osteogenesis in mice, leading to vertebral and cranial defects due to the down-regulation of crucial extracellular matrix proteins essential for bone formation [43].

The synergistic effects of *NELL-1* and bone morphogenetic proteins (BMPs) have been confirmed. Cowan et al. in 2007 investigated the synergistic effects of *BMP-2* and *NELL-1* on bone formation, demonstrating a robust synergistic impact on myoblasts both in vitro and in vivo. The results suggest that simultaneously stimulating both *NELL-1* and *BMP-2* could have greater therapeutic benefits and fewer negative side effects compared to only one pathway [32]. Another study also revealed this synergic effect in calvarial bone regeneration in vivo [44]. Moreover, research on the coding region of human *BMP9* and human *NELL-1*, revealed that *NELL-1* can be upregulated by *BMP9*, leading to the acceleration and enhancement of mineralization and maturity of *BMP9*-induced bone formation [45]. Considering that *BMPs* are crucial genes for bone and cartilage development [46], leveraging the synergistic effect of these genes with *NELL-1* could hold significant promise in the treatment of osteoporosis.

Gene therapy involving the use of *NELL-1* has shown promising outcomes in promoting bone regeneration. Li et al. investigated the treatment effects of *NELL-1* in a femoral defect model in rats. Three groups of treatments were established, each with eight rats, receiving either 1.5 mg/ml *NELL-1*, 0.6 mg/ml *NELL-1*, or phosphate-buffered saline as a Nell-free control. The results showed that both *NELL-1* -treated groups had more bone formation compared to the control group, and higher concentrations of *NELL-1* led to greater bone volume [33]. Similarly, Guo et al. investigated the treatment effect of *NELL-1* on osteolysis caused by polyethylene using a mouse model, finding that *NELL-1* promoted new bone growth, compensating for osteolysis better than control group [34].

*NELL-1* promotes osteoblast differentiation and mineralization, leading to increased bone formation. This is achieved by stimulating the expression of key osteogenic genes like *RUNX2* and *Osterix*, which are essential for osteoblast function. An investigation on human osteogenic sarcoma and primary human osteoblast cells the findings indicate that *Osterix* functions as a direct transcriptional regulator, suppressing *NELL-1* gene expression. This contributes to a fine-tuned balance of regulatory effects on *NELL-1* transcription in coordination with *Runx2*, potentially playing a critical role in osteoblast differentiation and mineralization [47].

*NELL-1* significantly increased the expression levels of various osteogenic genes and proteins, such as *ALP*, *OCN*, *Runx2*, *OPG*, *Col-I*, and Osterix. *NELL-1* can enhance the osteogenic differentiation of pre-osteoblasts on titanium surfaces by activating the mitogen-activated protein kinase/extracellular signal-regulated kinase signaling Pathway [30]. The activation of the *NELL-1* gene also promotes osteogenesis by regulating the *Runx2*/*Osterix* [31]. The binding between *NELL-1* and *APR3* is another pathway to enhance human osteoblast differentiation and mineralization [28].

In our study, rs1901945, rs1584851, and rs58028601 in the *NELL-1* were related to TBS. Several genome-wide association studies have identified SNPs within or near to this gene related to bone mineral density and osteoporosis [48–50]. These signals, along with the research findings that reveal the biological effect of the *NELL-1* gene on bone, indicate the potential impact of this gene on BMD and TBS.

We found that the GRS derived from SNPs of *NELL-1* was related to TBS. This suggests that GRS contributes significantly to explaining the variability in TBS levels beyond the other variables considered in the analysis. It is reasonable that the R2 values for the GRS in our study were relatively smaller compared to the other variables in the models. Despite this, the consistent higher R2 values in the models, including GRS underline its significance as a contributing factor to TBS. While the impact of GRS was small compared to other factors, it is still a significant factor for TBS.

Our study had some limitations. In this study, GRS was calculated using the three SNPs located on a single gene. Other limitation was the inability to compare our results with other studies due to the limited evidence about genes related to TBS, especially in a similar population. However, the present study was the first genetic investigation focusing on the TBS phenotype within the Iranian population.

## Conclusion

We investigated genetic variants of *NELL-*1 to test their relationships with TBS. Three SNPs were associated to TBS after adjusting for age and sex. *NELL-1* is primarily expressed in osteoblasts, the cells responsible for creating bone tissue. This gene plays a critical role in bone formation by promoting osteoblast differentiation and mineralization. Given that NELL-1 is primarily expressed in osteoblasts, targeting this gene for therapeutic interventions could hold promise for treating bone-related conditions such as TBS and osteoporosis. Utilizing genetic information for personalized medicine interventions based on individual genetic profiles could lead to more targeted and effective prevention, diagnosis, and treatment strategies to enhance bone health outcomes. Further research is essential to gain a deeper understanding of the genetic factors influencing TBS and to uncover additional genes that may be involved in regulating bone quality.

## Supporting information

S1 Data (DOCX)
