# Supplementary material for: Neural EGFL like 1 as a novel gene for Trabecular Bone Score in older adults: The Bushehr Elderly Health (BEH) program
Source: PLoS One. 2024 Sep 10;19(9):e0309401. doi: 10.1371/journal.pone.0309401 (PMC11386414; doi:10.1371/journal.pone.0309401)
Supplement: S1 Data — (DOCX) [file pone.0309401.s001.docx]

Data cannot be shared publicly because of ethical considerations. Data are available

from the School of Public Health & Allied Medical Sciences- Tehran University of

Medical Sciences Ethics Committee (contact via research

ethics Committees of School of Public Health & Allied Medical Sciences- Tehran

University of Medical Sciences, Email: [research@tums.ac.ir](mailto:research@tums.ac.ir) & [Ethics@sina.tums.ac.ir](mailto:Ethics@sina.tums.ac.ir), Tel: +98-81633619) for researchers who meet the criteria for access to confidential data.
